# Low field, high impact: democratizing MRI for clinical and research innovation

**DOI:** 10.1093/bjro/tzaf022

**Published:** 2025-09-25

**Authors:** Derek K Jones, Daniel C Alexander, Karen Chetcuti, Mara Cercignani, Kirsten A Donald, Mark A Griswold, Emre Kopanoglu, Ikeoluwa Lagunju, Johnes Obungoloch, Godwin Ogbole, Marco Palombo, Andrew G Webb

**Affiliations:** CUBRIC (Cardiff University Brain Research Imaging Centre), School of Psychology, Cardiff University, Cardiff, CF24 4HQ, United Kingdom; UCL Hawkes Institute and Department of Computer Science, University College London, London, WC1E 6BT, United Kingdom; Radiology Division, Department of Paediatrics and Child Health, Kamuzu University of Health Sciences, Blantyre, BT3, Malawi; CUBRIC (Cardiff University Brain Research Imaging Centre), School of Psychology, Cardiff University, Cardiff, CF24 4HQ, United Kingdom; Department of Paediatrics and Child Health, Red Cross War Memorial Children’s Hospital, University of Cape Town, Cape Town, 7701, South Africa; Neuroscience Institute, University of Cape Town, Cape Town, 7701, South Africa; Department of Radiology, Case Western Reserve University, Cleveland, OH, 44106, United States; CUBRIC (Cardiff University Brain Research Imaging Centre), School of Psychology, Cardiff University, Cardiff, CF24 4HQ, United Kingdom; Department of Paediatrics, College of Medicine, University of Ibadan, Ibadan, 200005, Nigeria; Department of Paediatrics, University College Hospital, Ibadan, 200005, Nigeria; Department of Biomedical Engineering, Mbarara University of Science and Technology, Mbarara, Uganda; Department of Radiology, University of Ibadan, Ibadan, 200005, Nigeria; CUBRIC (Cardiff University Brain Research Imaging Centre), School of Psychology, Cardiff University, Cardiff, CF24 4HQ, United Kingdom; School of Computer Science and Informatics, Cardiff University, Cardiff, CF24 4AG, United Kingdom; Department of Radiology, C.J.Gorter MRI Center, Leiden University Medical Center, Leiden, 2333 ZA, The Netherlands

**Keywords:** low-field, democratizing MRI, AI, image quality transfer, brain imaging, LMIC, Africa, neuroscience, scalable MRI, resource-limited settings

## Abstract

MRI is a cornerstone of modern clinical medicine and neuroscience, yet it remains largely inaccessible in low- and middle-income countries (LMICs) due to high costs, complex infrastructure requirements, the need for specialized personnel, and dependence on proprietary systems. Portable low-field MRI (LF-MRI), operating below 100 mT, offers a compelling alternative: low-cost, more accessible, and increasingly powerful, thanks to advances in hardware engineering, acquisition physics, image reconstruction, and open-source software. Reviewing and building upon recent progress, we, a multidisciplinary team of clinicians, physicists, engineers, and global health researchers based both in LMIC and HIC settings, present a formal argument for the adoption of LF-MRI as a catalyst for discovery science and healthcare innovation in LMICs. LF-MRI can produce clinically meaningful images and rich research data, enabling population-scale studies in neurodevelopment, ageing, and neurogenetics. But we argue that systems must be open, upgradeable, and co-developed, allowing potential for local teams to maintain, adapt, and scale technology according to their needs. Beyond the scanner, we outline the ecosystem required for success: data infrastructure, training pathways, ethical data governance, and equitable collaboration. We issue a call to researchers, vendors, and funders to reframe MRI as a globally accessible technology, capable of supporting diverse research agendas and delivering transformative health impact, particularly where it is needed most.

## Introduction

Over the last 30-40 years, MRI has transformed clinical medicine, offering unparalleled insights into tissue structure, function, and metabolism. Yet, this revolution has not had universal geographical reach. While high-income countries (HICs) have woven MRI as standard into clinical care and discovery science, many low- and middle-income countries (LMICs) still face critical shortages in equipment, expertise, and infrastructure.^1–5^ The result is a stark inequity: millions remain without essential diagnostic imaging, and large populations are underrepresented in neuroimaging research.^6^ This disparity is not due to a lack of innovation, but rather systemic barriers that have gone unchallenged for too long.^7^

In the late 20th century, there was notable innovation in commercially available low-field MRI (LF-MRI) systems, particularly those using large, fixed permanent magnets. For clarity, while definitions of “low field” vary across centres, here we focus on MRI systems operating at 100 mT (0.1T) or less, consistent with the emerging consensus from the International Society for Magnetic Resonance in Medicine (ISMRM) Low Field Study Group and Safety Committee.^8^ We refer to these as “LF-MRI” throughout this manuscript. These systems were applied across a range of applications, including brain, breast, and bone.^9–18^

However, enthusiasm for these LF-MRI systems waned as superconducting systems became available, offering higher field, increased spatial resolution, and faster scan times. This shift redirected commercial and research focus away from permanent magnet technologies, which had maximum field strengths ∼0.35T, and came with an increased purchase price and total cost of ownership, meaning that such systems became unaffordable in many parts of the world. Funding streams and industry support are increasingly aligned with the demands of high-income markets, where the focus was on maximizing image quality and throughput. As a result, commercial investment and scientific prestige became tightly linked to high-field systems, further marginalizing low-field innovation. So, as the community has since “moved on” to 1.5T, 3T or higher for radiological/research applications of MRI, there has been a tendency to dismiss LF-MRI. However, the outdated (and incorrect!) assumption that “low field means low quality means low utility” has hindered efforts to expand imaging access where it is most needed. We must challenge this mindset, especially within discovery science, where different measures of utility apply.

Today, we are witnessing a renewed interest in LF-MRI,^19^^,^^20^ driven primarily by a societal drive to expand imaging access globally. To reduce the total costs of ownership substantially and build sustainable clinical and research infrastructure, the only option is to use portable low-field devices. Fundamental technical advances in new high remanence magnetic materials, efficient MR pulse sequences, low-noise electronics, and image reconstruction techniques have together resulted in far higher quality images from such portable units than were possible using the large fixed permanent magnet systems in the 1990s.

Recent advances in MRI physics acquisition,^21^^,^^22^ image reconstruction,^23^^,^^24^ image enhancement,^22^^,^^25–35^ and noise-reduction/artifact handling^36–40^ have transformed the potential of LF-MRI. These new systems are being developed for a range of applications, from neuroimaging,^41–48^ to prostate,^49^^,^^50^ or even for whole body systems.^51^

As such, LF-MRI has real potential to significantly disrupt the *status quo* in global clinical and research imaging. LF-MRI is no longer merely a low-cost fallback; rather, it represents a powerful and versatile platform capable of delivering meaningful diagnostic and research insights, not only in settings where conventional high-field imaging is inaccessible, but also as a *complementary* tool in environments where it is accessible. As such, LF-MRI is poised to expand the utility of MRI across diverse clinical and geographic contexts. However, to realize the potential of LF-MRI, particularly in LMICs, technology alone is not enough. We must create a complete, robust ecosystem: developing not just the scanners, but the data infrastructures, the training pathways,^52^ and the research communities needed to sustain long-term impact, all suited to local environments.

A blueprint for addressing MRI accessibility in Africa already exists. For instance, an extensive survey was conducted on the current MRI facilities and needs across the African continent, with a framework recommended that integrates 4 core pillars of activities: advocacy, training, networking, and translational research innovation, to tackle Africa’s diverse MRI challenges.^4^ In another study,^53^ five key actions were identified to improve the situation: (1) enhancing low-field technology from the hardware, data acquisition, and image processing perspectives, leveraging recent advances in artificial intelligence (AI); (2) building physician confidence through local training programs; (3) prioritizing open-source approaches to foster sustainable local growth; (4) developing AI and data manipulation algorithms tailored to the needs of resource-limited communities, rather than relying on the power- and data-intensive models used in HICs; and (5) increasing funding from HICs to support sustainable growth in LMICs.

Building upon these foundations, we present a formal argument for the adoption of LF-MRI as a catalyst for discovery science and healthcare innovation in LMICs. Through this work, we seek to demonstrate how the unique advantages of LF-MRI—including its affordability, portability, and reduced infrastructural requirements—can significantly expand access to advanced diagnostic imaging, bridge critical gaps in healthcare delivery, and stimulate research and innovation in resource-limited settings. The majority of our examples are related to applications of LF-MRI in the brain, both in HICs and LMICs, reflecting current use cases.

Our goals are:

1. to review the substantial progress made in LF-MRI technology over the past few years and to anticipate the future developments that are likely to reshape the landscape of imaging in LMICs;
2. to challenge both the radiology and neuroscience communities to reconsider the scope of problems that are important in regions such as Africa, and which LF-MRI can address, and to critically define what technical advances are truly necessary to maximize its clinical utility and research potential; and
3. to issue a strong and urgent call to industry partners: to support the creation of a sustainable, accessible, and open global infrastructure by endorsing the certification, refinement, and dissemination of open-source LF-MRI systems. We firmly believe that these systems hold potential to meaningfully improve health outcomes in LMICs, and their success will depend on collective efforts that prioritize innovation, inclusivity, and long-term global impact over narrow commercial interests.

In pursuing these objectives, we aim not merely to highlight the promise of LF-MRI, but to catalyse a movement that transforms how imaging technology is developed, deployed, and leveraged for public good across the world. We envision LF-MRI systems designed with open-source hardware and software—affordable, serviceable, scalable, and flexible enough to support neuroscience research in diverse settings. These systems will enable experimental protocols, large-scale population imaging, and novel scientific discoveries beyond the constraints of traditional workflows. This paper is a call to action: to rethink, to reinvent, and to extend the frontier of MRI to every corner of the world.

While our recommendations are grounded in the urgent and unmet needs of LMICs, the challenges and opportunities described here apply broadly. Underserved and remote regions in HICs often face similar structural limitations in access to MRI. The arguments we make for low-cost, flexible imaging systems, centred around openness, scalability, and local leadership, are just as relevant across diverse global contexts.

This review is structured around 6 key themes: the first section—Why? Defining the scale of the access gap; the second section—What? Examining “Conventional MRI” and why it is just not delivering in LMICs; the third section—How? (Part 1): Technical innovations that make low-field systems viable for LMICs; the fourth section—How? (Part 2): Discussing the supporting infrastructure and training required; the fifth section—A Call to Action: Issuing a call to coordinated, collective action; and the sixth section—Conclusions: We provide concrete steps towards equitable global imaging, focusing on an African context, but the lessons apply equally to other areas of research and geographical locations such as South America and South-East Asia.

## Why? Defining the scale of the access gap

### The global MRI divide

LMICs carry over 70% of the world’s disease burden,^54–58^ yet generate only a small percentage of the world’s neuroimaging publications. This reflects not only a shortage of access to fully operational scanners but also a broader ecosystem gap: limited imaging-trained personnel,^7^^,^^59^ unaffordable (and therefore scarce) service contracts, and underdeveloped data infrastructures. In 2018, a total of 84 MRI scanners served a combined population of 372.6 million across 16 countries in West Africa—equivalent to just 1.1 scanners per 5 million people.^1^ By contrast, the United States hosts approximately 196 scanners per 5 million, while Japan has 276 scanners per 5 million people (https://bit.ly/OECD-MRI-Units).

This represents 178-fold discrepancy in scanner availability, which has a significant impact: delayed diagnoses, missed opportunities for targeted treatment, and a skewed evidence base that underrepresents populations with high genetic diversity and distinct disease burdens.^6^^,^^60^^,^^61^ This is not just about a challenge of access to MRI scanners; we are missing out on a huge opportunity to address health equity, to establish research leadership, and for scientific advancement.

### Missed potential for diagnosis and discovery

Neurological conditions—from stroke to neurodevelopmental and neurodegenerative disorders—often go undiagnosed (or have a delayed diagnosis) in LMICs due to a lack of access to services, including imaging. Clinical decisions are made without the benefit of visualization, leading to suboptimal outcomes. Yet, over the last few years, LF-MRI systems have shown increasing clinical value in a whole host of diseases/clinical conditions. For example, in: cerebrovascular disease and stroke^62–66^; haemorrhage^67^^,^^68^; post-cardiac arrest^69^; white matter hyperintensities^70^; demyelinating diseases^71–73^; optic neuritis^74^; multifocal leukoencephalopathy^75^; epilepsy^76^^,^^77^; dementia^65^^,^^78^; intracranial midline shift^79^; hydrocephalus^80–82^; and malaria.^83^ LF-MRI systems also make it possible to scan patients who have contra-indications to being scanned—on high-field systems, from a safety perspective, or in which the images are likely to be less helpful for diagnostic use due to severe image artefacts.

The literature contains reports of scanning patients with LF-MRI: with implanted cardiac devices^84^^,^^85^; undergoing ECMO (Extracorporeal Membrane Oxygenation)^86–91^; with an intracranial bolt^92^; with cochlear implants.^93^ While full testing at the specific field strength is still required for any implant to be considered safe, even at lower field strengths,^94^ it is reasonable to assume that LF-MRI systems can widen access to imaging for critically ill patients with implants.

While clinical utility is one benefit, LF-MRI also represents an opportunity to expand the frontiers of neuroscience research. By enabling imaging in communities historically excluded from MRI-based studies, LF-MRI platforms can unlock insights into brain development, ageing, and disease across vastly underrepresented population.^95^ Recent analyses, such as those commissioned by the Wellcome Trust,^6^ have emphasized that African neuroscience holds a globally unique value proposition. Africa’s unparalleled genetic diversity, distinct ecosystems, and unique exposure to neurological risk factors such as infection, trauma, and malnutrition offer opportunities for breakthrough discoveries in brain health, neurodevelopment, and disease resilience. Importantly, fostering research infrastructure in these settings is not only about equity but also about scientific necessity: without greater inclusion of African populations, our models of brain health will remain grossly incomplete.^96^ LMIC researchers are underrepresented in datasets, grant funding, and authorship, stunting the development of local, contextually relevant research agendas. As highlighted in the *Showcasing African Neuroscience* initiative,^6^ capacity building must go beyond installing scanners; it must involve co-developing research agendas, building local innovation pipelines, and embedding African scientists into the global discovery ecosystem.

Thus, the missed opportunity is 2-fold: patients miss out on diagnosis and treatment, and science is less global than it should be. But, with the establishment of appropriate imaging ecosystems, these settings could become powerful catalysts for a truly global and inclusive discovery science—especially in fields like neurogenetics, paediatrics, and infectious disease neurology.

## What? “Conventional MRI” is just not delivering in LMICs

### The reality of high-field MRI

MRI’s diagnostic value is without question. However, standard clinical-field systems (1.5T or 3T) commonly used in HICs are often just not suitable for LMIC settings. First, they are expensive to purchase. But there are additional associated costs, such as the need for an electromagnetically shielded room, climate-controlled facilities, cryogenic gases, and electricity costs. On top of that, and perhaps most crippling, is the prohibitive maintenance contracts imposed by the vendors. Overall, the 10-year total cost of ownership is many multiples of the original purchase price. Much too often, MRI systems arrive at a site with short-term initial support packages and no long-term sustainability strategy. Once that initial service contract expires, institutions struggle to afford ongoing maintenance and other costs that are not always apparent at the outset. The result? Non-functional systems that ultimately get relegated to the “medical equipment graveyards.” This pattern has led to hesitancy about the viability and concern about the financial risk associated with commercial MRI equipment in public health systems already under strain, deterring further investment and undermining trust in MRI as a viable technology.

The challenges go well beyond affordability. As Jha notes,^97^ market uncertainty—not just economic status—often limits investment in Africa’s MRI ecosystem. Vendors prefer predictable returns, making long-term planning difficult and sustainable development elusive. Even when institutions can cover costs, procurement decisions based solely on price—rather than service quality—often lead to poor maintenance contracts and prolonged downtimes.^98^ This all, clearly, must change.

### Where current low-field models fall short

LF-MRI presents a fundamentally different opportunity. In addition to the emerging clinical applications highlighted earlier, these systems are smaller, often mobile, require minimal shielding, and consume significantly less power than conventional high-field scanners. However, despite these potential advantages, the adoption of LF-MRI systems in LMICs has remained limited. This is due to several reasons. First, many commercially available LF-MRI systems deployed in LMICs were originally designed for high-income healthcare environments. These systems often have high acquisition costs (eg, >USD 300 000) and require ongoing proprietary maintenance contracts, making them financially unsustainable in LMIC settings—similar to the limitations seen with commercial high-field MRI systems.

Second, an important design feature for the widespread adoption of LF-MRI systems in LMIC settings is *full portability*. In our view, the term “portable” is interpreted inconsistently across the literature. Notably, 37 of the references in the bibliography of this article use the word *portable* in their titles, yet the systems described differ greatly in their actual ability to be transported. For clarity, we propose the following 3-category classification for MRI systems:

- *Fixed*—systems that are semi-permanently integrated into a building’s infrastructure, typically housed within a Faraday cage and connected to fixed plumbing, electrical, and refrigerant supplies.
- *Mobile*—systems that can be wheeled or otherwise moved between locations without requiring a shielded room or fixed infrastructure but require some form of motorized transport.
- *Portable*—systems meeting the Oxford English Dictionary (OED) definition: “Capable of being carried by hand or on the person; capable of being moved from place to place; easily carried or conveyed. Also: (designating a device, apparatus, etc) made smaller and lighter than normal, to enable it to be carried easily.”

A *fully portable* system is not only independent of shielded rooms and fixed infrastructure but also light enough to be manually transported—by one or more people—into any part of a hospital or research building.

This distinction is important because mobility alone does not guarantee deployment at the point of need, particularly in low-resource hospitals where elevators, wide corridors, or ramps may be absent. We have encountered situations where a nominally portable system, even when provided with a motorized drive, could not be moved between floors due to a lack of elevators, or across steep ramps to reach critical care areas. In such contexts, genuinely portable systems—meeting the OED definition—could be transformative for expanding access to imaging.

Third, to overcome the technical limitations of LF-MRI, such as low signal-to-noise ratio (SNR), relatively coarse spatial resolution (∼2-3 mm), long scan times, and lack of tissue contrast, innovation is crucial. As SNR scales with field strength, LF-MRI is typically associated with low SNR, which is often compensated for with coarser spatial resolution and/or longer scan times. Additionally, LF-MRI tends to suffer from reduced tissue contrast, further complicating imaging in certain applications. To address these challenges, cutting-edge solutions like AI-based approaches,^22^^,^^25–34^ fast imaging sequences,^21^^,^^22^ and innovative reconstruction techniques^23^^,^^24^ are key. Still, they can only be fully leveraged if researchers have access to the necessary data and flexibility to explore these options. Commercial LF-MRI platforms are often closed to user programming, with vendors citing proprietary considerations. This can limit flexibility in settings where users seek access to raw (k-space) data for developing alternative reconstruction techniques, applying advanced denoising algorithms, or customizing pulse sequences. While it is possible—through negotiation and formal agreements—to gain such access from both LF and high-field vendors, these processes can be time-consuming and are not always feasible for institutions with limited resources. Our intention is not to generalize, but rather to emphasize the importance of lowering barriers to innovation and encouraging broader access to development tools, especially in LMIC contexts where such access can be transformative. While there is huge momentum gathering within the LF-MRI physics and engineering community to help advance MRI technology in LMIC settings, without access to these essential facets of MRI development—such as raw data and customizable programming options—the opportunities for contributing meaningfully to the advancement of LF-MRI in LMICs are severely constrained.

Flexibility is essential. Research-grade LF-MRI systems must provide full user-level control over acquisition and reconstruction pipelines, rather than enforcing vendor-locked workflows. To truly democratize MRI, foster innovation, and embed users within global collaborative networks, LF-MRI systems must be developed according to open-source principles. Systems designed with modular hardware, openly shared construction plans, and community-driven software become locally maintainable, adaptable, and scalable. Crucially, this would enable institutions in LMICs to take ownership of their imaging infrastructure. Free from reliance on proprietary systems and international vendors, local teams can perform their own maintenance and upgrades—and even build scanners onsite.^99^ Just as importantly, open and modular designs support upgradeability: the ability not only to sustain current systems, but also to expand their capabilities over time. This shift from passive maintenance to active innovation is essential for building an imaging ecosystem that achieves true independence from HIC supply chains.

That said, open access is not appropriate in all settings. In both high- and low-resource environments, there are important use cases—such as routine clinical deployments—where streamlined, locked-down systems are more suitable. These minimize training demands, reduce risk of error, and accelerate adoption. Indeed, many successful research studies and clinical initiatives have relied on such systems to deliver critical results.

Our argument is therefore not that all systems should be fully open, but that the option for full access should exist for research environments in LMICs just as it does in HICs. However, such access for LMICs should not be accompanied by very expensive and restrictive research contrasts, as are common in HIC settings. This ensures equity in the ability to innovate and adapt, while also supporting the parallel deployment of user-friendly, closed systems for routine imaging and clinical care.

As with any advanced tool, flexibility must be paired with safeguards: structured training, clear usage policies, and measures to protect image quality and patient safety. In the long term, even if some systems continue to operate in locked mode to ensure reliability in the field, control of these systems should rest with trained local personnel—once the necessary expertise has been established through relevant training—thereby ensuring independence and sustainable operation. It is this combination of accessibility and responsibility that will enable LF-MRI platforms to meet the full spectrum of imaging needs across global contexts.

By prioritizing affordability, ease of maintenance, and adaptability to local conditions, such systems promise to enhance sustainability and expand access to advanced diagnostic imaging in underserved regions. A real concern is that without such a shift in approach, these commercial systems risk repeating the same failures that plagued earlier high-field deployments: being underutilized or, worse, ultimately joining the ranks of abandoned equipment. The future lies in investing in tailored, modular, open-access LF-MRI platforms—designed from the outset for clinical relevance, affordability, maintainability, and research-readiness in LMICs.

## How? (Part 1): designing MRI systems that work for LMICs

### Reframing the narrative: low field ≠ low quality

It is high time that we dispel the myth that lower field strength inherently equates to lower clinical or research utility. Yes, SNR is indeed lower at reduced field strengths if everything else is held constant,^8^^,^^100–104^ but it is important to recognize that in many high-field settings (eg, 1.5T or 3T), the available SNR far exceeds what is necessary for effective diagnosis of many diseases. Instead of chasing after higher image quality, we implore the community to shift their focus to whether an image is *fit for purpose*, where one could consider “scanning with less.”^105^ In this regard, LF-MRI is already delivering (see the section “Missed potential for diagnosis and discovery”)—particularly for conditions where even reduced contrast is sufficient or where repeated scanning, context-specific markers, or broader accessibility are more important than peak resolution.

In addition, recent advances in acquisition physics, image reconstruction, and AI-based image enhancement mean that we can produce diagnostically useful and research-enabling images in settings where high-field MRI is impractical. But we also don’t have to do identical things to what we do at higher fields. With smart design and advanced software, LF-MRI does not have to be adopted as a compromise, or even just as a clinical diagnostic tool, but as a platform that enables research and innovation and allows people to be imaged in places that were just not previously possible.

### Boosting performance through advanced acquisition and reconstruction

Clinicians and researchers have become accustomed to using fast acquisition techniques like parallel imaging to reduce the total scan time per patient, which are often unavailable or less effective on current LF-MRI systems. Additionally, certain imaging modalities that are particularly susceptible to low SNR, such as diffusion MRI, have traditionally relied on multiple signal averages to compensate, further extending scan times. Therefore, it is crucial to think creatively about how LF-MRI systems are used and what imaging targets are prioritized, especially if reliable measures are to be obtained within tolerable acquisition times.

When designing pulse sequences for LF-MRI, it is crucial to recognize that approaches considered optimal at high-field strengths may not necessarily translate well to low-field environments. For example, sequences such as echo planar imaging (EPI), which rely heavily on high magnetic field homogeneity and rapid gradient switching, are less feasible at LF due to inherently lower field homogeneity due to their smaller size and limitations in gradient performance due to the simplified architecture, necessitating other approaches.^106^^,^^107^ Conversely, sequences traditionally constrained at high field due to specific absorption rate (SAR) considerations,^108^^,^^109^ such as T2 fast spin echo (T2-FSE) become much more practical at low field, where SAR is significantly reduced.^110^ Another important consideration is the choice between 2D and 3D acquisitions: while 2D imaging is often preferred at high field for its speed and reduced artefact burden, 3D acquisitions may offer better SNR efficiency at low field, helping to compensate for the inherently lower signal levels.^41^ Thus, careful adaptation rather than direct translation of high-field sequence strategies is essential for optimizing LF-MRI performance.

We feel it is important to make the distinction between high-throughput clinical applications—where efficient protocols including T_1_-weighted, T_2_-weighted, FLAIR, and basic diffusion imaging are essential—and research-focused applications, where longer acquisitions may be acceptable because participants volunteer for study purposes. This distinction should form part of the broader discussion about LF-MRI capabilities. Expectations must also be reframed: if it takes twice as long to collect high-quality data at low field compared to 3T, but the scanning is taking place in a setting where access to 3T is either not feasible or contraindicated (as discussed earlier), then the LF-MRI solution is not merely a compromise.

While longer scan times do increase the risk of motion artefacts, innovations in motion correction techniques at low field^111^ are helping to mitigate these issues, making prolonged acquisitions more manageable. However, recent developments have also facilitated the acceleration of scanning. Compressed sensing, sparse sampling, and machine learning-based methods allow high-quality imaging from fast, under-sampled LF-MRI acquisitions.^23^^,^^112^^,^^113^

AI techniques such as Image Quality Transfer and *SynthSR*, which learn mappings from images with low information content (eg, typical low-field images) to those with higher information content (eg, typical high-field images), enable LF-MRI images to approximate contrasts traditionally available at high-field.^22^^,^^25–34^ They can aid conspicuity of subtle features^27^^,^^28^ and familiarity for a radiologist used to high-field images, as well as enable standard image processing pipelines to produce familiar imaging biomarkers. Emerging techniques like Magnetic Resonance Fingerprinting^114^ offer powerful, time-efficient ways to extract quantitative biomarkers—even at low-field.^115–118^

Multiple MR images are often acquired together because they offer complementary diagnostic information, which are then processed separately in standard reconstruction pipelines. Joint reconstruction techniques exploit the underlying similarities across accelerated acquisitions with different contrasts of the anatomy, significantly enhancing SNR and revealing details that would otherwise be buried under noise while still faithfully preserving each image’s unique contrast.^119^ These SNR improvements can be further amplified by optimizing MRI acquisition protocols holistically, rather than optimizing sequences in isolation.^120^

Finally, to enhance the research capabilities of LF-MRI systems, particularly for “SNR-hungry” contrasts like diffusion MRI, increasing the available gradient amplitude may be essential. In diffusion imaging, the degree of diffusion weighting is quantified by the *b*-value, which scales with the square of the gradient amplitude and the duration of the diffusion gradients.^121^^,^^122^ While one way to increase *b*-value is to lengthen gradient duration, this also increases the echo time (TE), which can reduce SNR due to T_2_ signal decay—an especially significant concern at low field. Therefore, higher gradient amplitude becomes critically valuable: it enables stronger diffusion weighting (higher *b*-values) without needing to extend TE, preserving more signal. This trade-off is particularly important for techniques like diffusion tensor imaging (DTI) at *b* = 1000 s/mm^2^, and even more so for advanced models like diffusion kurtosis imaging (DKI), which offer greater sensitivity to tissue microstructure and pathology than DTI,^123–131^ but require higher *b*-values.

However, it is crucial to ensure that increased gradient strengths do not overlook key considerations. The cost of amplifiers used to drive these high-power gradients should not significantly inflate the price of the LF-MRI system. Additionally, the power requirements must not impose a heavy financial burden on institutions, especially in regions where electricity costs are high. Another key challenge to consider is that LF-MRI systems generally lack integrated cooling systems, without which, system heating may lead to performance drift, potentially requiring longer acquisition times to manage this issue. This problem is known to be more pronounced in LMICs, particularly in equatorial regions with higher ambient temperatures, where climate-controlled environments are not always available.

Therefore, more research is needed to explore the limits of practical, low-cost, sustainable, and stable high-amplitude gradient implementations in LF-MRI systems.^132^ Ultimately, necessity drives innovation, and LF-MRI presents an opportunity to rethink and optimize imaging strategies rather than simply applying high-field expectations to low-field systems.

### Serving radiology and discovery science

With the right protocols, LF-MRI can produce not only clinically useful images but also rich, high-quality data suitable for advanced research applications. Recent advances in physics and modelling are further expanding the reach of LF-MRI. For neuroscience, this opens up many possibilities, including DTI and tractography,^133^^,^^134^ diffusion-based spherical deconvolution and connectomics,^135^^,^^136^ tractometry,^137^ quantitative relaxometry,^113^^,^^116^^,^^138–141^ magnetization transfer,^142^^,^^143^ magnetic resonance spectroscopy,^144^ and angiography^145^^,^^146^—even at low field. Crucially, we are not trying to replicate the visual appearance or spatial resolution of 3T MRI, but to develop alternative imaging biomarkers that can function within this emerging paradigm. Importantly, the affordability and scalability of LF-MRI make it uniquely suited for large-scale population studies, supporting advanced imaging across diverse contexts including paediatric neurodevelopment, ageing and neurodegenerative diseases.

It is well established that the best healthcare environments are those embedded within active research ecosystems. Scientific innovation and clinical excellence go hand-in-hand: hospitals with active MRI research programs not only advance knowledge but also attract and retain skilled professionals, deploy the latest protocols, and optimize patient care pathways. LF-MRI offers a foundation on which to build this synergy of innovation and impact in LMIC settings.

To realize their full potential, research platforms must be *fully open-source design*, *co-developed*, and *adaptable*. Neuroimaging research demands flexibility, and the highest impact will come when innovations are embedded in platforms that can evolve alongside local and research priorities.

By “open-source,” we mean transparent access to the scanner’s control software, sequence programming environment, and, where feasible, aspects of the hardware design. This includes the ability to adjust key parameters—such as gradient timing and radiofrequency (RF) pulse shapes—implement new pulse sequences via a sequence development kit, and access image reconstruction code so that local teams can optimize, maintain, and extend the platform according to evolving needs.

This level of openness is not proposed without safeguards. As with advanced research platforms in HICs, full access should only be granted following structured training in MRI safety, system design, and basic physics. We envision these systems being deployed in research environments and supported by dedicated education and training programmes—many of which are already in development through our wider collaborative network.

Enabling local teams to maintain and adapt these systems mirrors established practice in many LMICs, where trained engineers already service complex diagnostic tools such as X-ray and ultrasound machines. Our goal is to foster equivalent technical capacity for MRI, ensuring long-term sustainability and independence.

While commercial systems that lock down interfaces or employ proprietary, AI-driven reconstruction pipelines can offer operational efficiency, they may also limit understanding and innovation. In research settings, especially LMIC researchers should have the same opportunities for exploration and optimization as their counterparts in HICs. Equitable access to open systems is therefore a crucial step towards addressing this imbalance.

Additionally, LMICs present unmatched opportunities for novel cohort studies in neurogenetics and environmental neuroscience—areas where population diversity is a scientific strength. Open-source LF-MRI systems can catalyse this research at scale, removing the logistical and financial barriers that come with more conventional imaging systems.

## How? (Part 2): building the ecosystem around LF-MRI

### Data infrastructure that fits the context

Conventional cloud-based infrastructures rely on stable internet and reliable electricity—conditions often absent in LMICs, particularly in sub-Saharan Africa. For instance, over 85 million people in Nigeria lack grid access,^147^ and inconsistent power severely limits the viability of “cloud-first” medical imaging strategies. To address this, data solutions must be tailored to local realities. These include on-device processing, compression at the point of acquisition, and hybrid storage models that reduce reliance on bandwidth-intensive cloud workflows. In environments with variable connectivity and frequent outages, battery-backed or solar-supported systems with voltage regulation become essential.^148^ Many LMIC institutions adopt a dual-system approach, using both offline and online infrastructure to bridge digital health gaps. Yet limitations persist: outdated hardware, limited capacity for long-term data archiving, and insufficient technical support hinder sustainable research programs. Innovations must incorporate offline-first design, delayed upload protocols, and local mirroring to ensure resilience and operational continuity.

Although advances in deep learning (DL) offer promise, many current models prioritize performance metrics like accuracy over clinical utility. High computational and energy demands render these DL approaches unsuitable for low-resource environments.^149^ Additionally, unreliable connectivity and limited training in cloud tools push many institutions towards on-premise processing despite the potential benefits of cloud-based systems.

To bridge these gaps, several priorities must be addressed:

- *Develop lightweight, energy-efficient AI models* tailored to LMIC settings.^150^
- *Build local capacity* through training in cloud resource use and digital infrastructure. Initiatives like AFRICAI (https://africai.org/) provide successful examples.
- *Invest in robust, scalable storage* and connectivity to reduce dependence on fragile physical media.

### Responsible data sharing for LF-MRI in LMICs

Global collaboration is essential for advancing LF-MRI, but it must be grounded in ethical data handling that protects local autonomy and ensures equitable participation. Federated data architectures and LMIC-led governance models offer a powerful pathway—enabling cross-site analysis without compromising local control, context, or benefit sharing.

#### Ethical governance and local ownership

Responsible data sharing in LMICs must address persistent ethical and equity challenges. Many countries lack robust legal frameworks to protect patient data, exposing it to risks of misuse or unauthorized transfer. Informed consent procedures must therefore be culturally adapted to clarify both local and international data use.

Adopting governance models aligned with the Global Alliance for Genomics and Health (https://www.ga4gh.org) promotes data sovereignty and equitable benefit sharing. Local Institutional Review Boards (IRBs) should oversee consent and review, while clearly defined Memoranda of Understanding can codify ownership, intellectual property rights, co-authorship, royalties, and technology transfer agreements (https://doi.org/10.1332/27523349Y2023D000000002).^151^ To further counter historical inequities in data exploitation, data-sharing agreements should guarantee transparent authorship and ensure that LMIC investigators are co-leaders—not merely data providers.

#### Federated learning as a privacy-preserving model

Federated learning (FL) has emerged as a transformative solution for collaborative AI development across institutions while preserving data privacy. Rather than centralizing data, FL enables each site to retain control over patient data and share only model updates (eg, gradients), significantly reducing privacy risks.^152^

A landmark FL study across 71 sites on 6 continents improved glioblastoma boundary detection by 33% compared to centralized models.^153^^,^^154^ Adapting this approach for LF-MRI in Africa allows models to learn from diverse scanner types and patient populations, enhancing generalizability across both high- and low-field systems,^155^ without sacrificing data ownership and confidentiality.

#### Technical infrastructure for federated collaboration

For FL to succeed in resource-constrained settings, robust, context-sensitive technical infrastructure is essential:

1. *Secure FL Clients:* Lightweight, containerized software that runs on local servers or workstations.^156^
2. *Encrypted Communication:* Homomorphic encryption and secure aggregation protect model updates in transit.^157^
3. *Data Harmonization Pipelines:* Standardized preprocessing for bias correction, denoising, and spatial normalization ensures interoperability
4. *Federated Orchestration:* A central server coordinates model aggregation and tracks convergence.^158^
5. *Offline-Compatible Tools:* Simplified, local Digital Imaging and Communications in Medicine (DICOM) anonymizers and edge computing solutions reduce dependence on cloud infrastructure while preserving essential metadata.

#### Training in FL and sustainability

Sustainable data sharing hinges on empowering local teams. This includes training in FL implementation, LF-MRI physics, and data governance.^159^ Establishing site-specific “FL Champions” ensures local maintenance and long-term autonomy.^154^

### Capacity building

A core challenge is the shortage of trained radiographers, radiologists, engineers, computer scientists, and physicists. International courses are often inaccessible due to cost or geography. But regional initiatives, such as the ISMRM, ISMRT Future Leaders, CAMERA,^59^^,^^160^ SMARTA,^7^^,^^161^ the ISMRM African Chapter,^162^ and RAD-AID^163–167^ are starting to address this through practical, inclusive training that builds lasting capacity. From the hardware and software side, there have been an increasing number of hands-on workshops (Mbarara, New York, Cape Town, Singapore), often in the shape of hackathons,^168^ where groups have constructed their own MRI systems based on open-source designs. Societies such as MICCAI have begun to issue image processing challenges based specifically on the reconstruction of low-field data (https://zenodo.org/records/15259777; https://zenodo.org/records/15081583). This starts to tap into a huge wealth of talent and enthusiasm in computing and AI in LMICs that can potentially be brought to bear on image analysis problems. Dedicated student training pipelines, integrated with LF-MRI initiatives, can empower the next generation of LMIC-based neuroscientists to lead discovery science. Programs must support research design, data analysis, imaging physics, and interdisciplinary skills, ensuring that students are not merely end-users of imported technology but active contributors to the global advance of discovery science.

### Knowledge without paywalls

Access to tools and literature remains a significant barrier to progress, especially for researchers and practitioners in LMICs. Strategies may include expanding open-access publishing and free software platforms, making conference attendance and the associated content more affordable. It is imperative that no radiologist or researcher is excluded from these vital resources due to a lack of institutional financial support. One of the most pressing concerns is the practice of charging publication fees for articles. Researchers in LMICs often face insurmountable financial barriers to publishing their work because of these fees, which are simply beyond their reach. This exclusion from the global research ecosystem is not just an issue of fairness; it means we miss out on valuable insights and perspectives that could drive innovation and progress. Without the participation of LMIC researchers, we may never fully understand the challenges and successes of deploying technologies like LF-MRI in these settings. By removing these financial barriers, we ensure that the contributions of those in LMICs are included, their experiences are heard, and the global research community can benefit from their unique perspectives. This is essential for advancing knowledge and improving healthcare systems globally. As discussed in the section “How (Part 1): designing MRI systems that work for LMICs”, technical barriers—such as locked-down systems that prevent pulse sequence modification or reconstruction customization—can stifle innovation and prevent collaboration. True research enablement demands open systems that support local scientific inquiry, not just clinical diagnostics.

## A call to action: when and what next?

### When?

The global health landscape stands at a critical juncture. Technological advancements in open-source MRI hardware, cloud computing, and image reconstruction algorithms have converged with growing LMIC-led innovation and an intensified international focus on health equity.^1^ High-quality neuroimaging is no longer acceptable to remain the exclusive privilege of HICs. Neurological conditions, including stroke, dementia, central nervous system infections, cancers, and epilepsy, impose a disproportionate burden on LMICs. Yet, imaging technologies that could transform diagnosis and care remain out of reach for many.^169^

We must challenge the entrenched paradigms of technological development that prioritize marginal gains in ultra-high-field systems over widespread accessibility. Innovations at 7T and 3T must not blind the global scientific community to the possibilities of lower-field MRI and profound clinical impact. Rather than viewing LF-MRI as a compromise, lamenting the absence of sub-millimetre resolution, researchers and clinicians are encouraged to consider it as an *additional frontier*—a complementary domain for innovation—where imaging systems, though less conventional, offer access to vital diagnostic and research insights. These systems can transform how we study populations and conditions historically excluded from advanced imaging. The goal is not to displace high-end labs, but to broaden the spectrum of innovation for impactful research. There remains a critical need for more scientists who are willing to apply their technical expertise to imaging systems that, while perhaps less conventional or refined by high-income standards, can nevertheless deliver essential diagnostic information in settings where such access has traditionally been unavailable. By adapting, innovating, and maximizing available tools and by building equitable, collaborative partnerships across different research environments, we can broaden the impact of LF-MRI.

Based on our own experience working in and/or extensive collaboration with colleagues in LMICs, there is a clear and growing call not for short-term charity or isolated donations, but for equitable partnerships, sustainable pricing models, and meaningful inclusion in leadership and decision-making processes.

Fee reductions alone are not enough. We must move beyond a “donate-and-forget” culture that, while often well-intentioned, delivers limited and short-lived impact. Instead, lasting progress will come through long-term collaborations that prioritize co-development, capacity building, and locally led innovation. This means designing, deploying, and maintaining imaging systems tailored to the realities and strengths of resource-constrained environments—systems that not only meet clinical needs but also unlock new research opportunities. Moreover, early investment in open, modular systems could unlock new markets, catalyse regional manufacturing and service ecosystems, and yield insights from populations never previously studied at scale. By supporting scalable, certification-ready systems, industry and philanthropy can enable both equitable impact and sustainable growth. All of this is not simply a future opportunity, but a responsibility that we need to act on now.

### Roadmap: people, platforms, and partnerships

**Figure 1.**
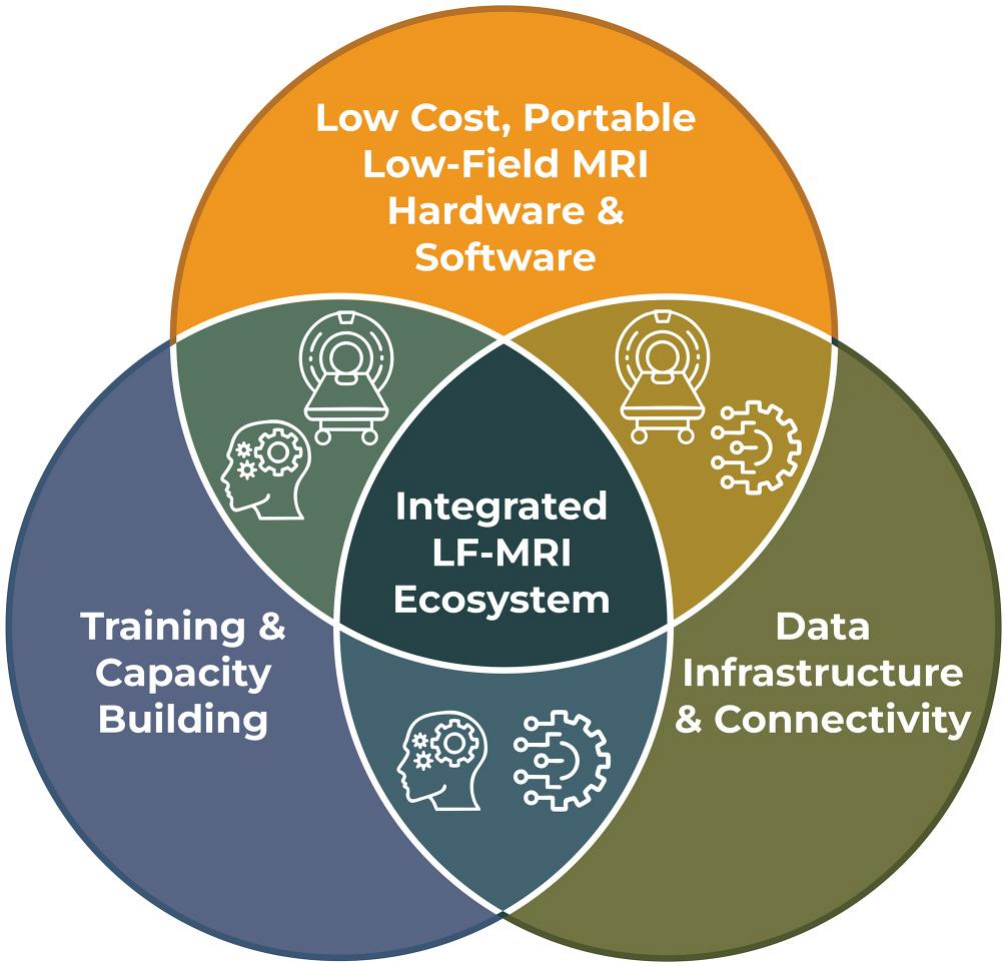
Integrated LF-MRI ecosystem: *People*, *Platforms*, and *Partnerships*. The foundational pillars required for the scalable and equitable deployment of LF-MRI in LMICs. *People*: Focuses on empowering local leadership and innovation. It emphasizes LF-MRI as a frontier for discovery tailored to LMIC health needs, fostering global networks, and building capacity through open-access training, mentorship, and inclusive representation in scientific governance. *Platforms*: Encompasses the design of sustainable, open-source MRI systems with modular hardware, accessible data. It includes infrastructure for ethical data governance, scalable storage and analysis, and regional engineering hubs to support maintenance and innovation. *Partnerships*: Advocates for inclusive collaboration between LMIC institutions, industry, and philanthropic organizations. It calls for co-development of certified systems, investment in scalable deployment, and recognition of LF-MRI’s role beyond clinical settings. Abbreviations: LF-MRI = low-field MRI; LMICs = low- and middle-income countries.

High-impact expansion of LF-MRI in LMICs demands coordinated action across 3 pillars: *People*, *Platforms*, and *Partnerships* (Figure 1), each reinforcing the others to build capacity, drive open innovation, and foster equitable collaboration.

#### People: empower local leadership and innovation

- *Embrace LF-MRI as a Frontier for Innovation:* Encourage clinicians, scientists, and engineers, particularly in LMICs, to see LF-MRI not as a fallback, but as a catalyst for discovery tailored to local health needs.
- *Connect with Global Networks:* Platforms like CAMERA, SMART-Africa, and the ISMRM African Chapter offer pathways for collaboration, mentorship, and visibility. The latter is a compelling example. Since its formation, the ISMRM African Chapter has fostered regional collaboration and training, helping position African scientists as leaders in MRI innovation. Its grassroots structure and sustained growth show how locally led platforms can catalyse long-term change.^4^
- *Build Local Capacity:* Expand training beyond scanning to include sequence development, image reconstruction, maintenance, quality control, and system optimization. Foster technical and scientific leadership through open-access training, hands-on workshops, and bilateral mentorship. Where possible, conference organizers, journal publishers, and training providers must adopt dynamic, means-based, or sliding-scale pricing models to ensure financial accessibility for LMIC researchers.
- *Promote Equitable Scientific Exchange:* LMIC researchers must be seen—and supported—as global contributors. This includes representation on editorial boards, funding panels, and policy bodies. Frameworks like *Africa Charter for Transformative Research Collaborations* (https://parc.bristol.ac.uk/africa-charter) and associated commentaries^151^^,^^159^^,^^170^ offer guidance. Representation in editorial boards, conference committees, policy bodies, grant review panels, and leadership roles must be institutionalized to ensure that LMIC leadership shapes the future of neuroimaging.

#### Platforms: design systems for sustainable discovery

- *Enable Open and Flexible MRI:* Advocate for open-source LF-MRI systems with modular hardware, open codebases, and access to raw data for local innovation.
- *Support Local Maintenance Ecosystems:* Establish regional engineering hubs to reduce reliance on international service contracts.
- *Build Smart Data Infrastructure:* Invest in secure, locally appropriate storage, compression, and cloud access to support remote mentoring, decentralized analysis, and real-time collaboration.
- *Develop Ethical Data Governance:* Support federated, LMIC-led frameworks to ensure ethical sharing and local data sovereignty.
- *Plan for Scale:* Infrastructure must support longitudinal and large cohort studies, including neurodevelopment, ageing, and environmental health—aligned with initiatives like UNITY.^171^
- *Address Certification and Standardization:* Open, modular systems allow for adaptation to local contexts but also pose challenges for regulatory approval. We must explore collaborative, scalable approaches to system certification that ensure safety and trust without stifling local innovation.

#### Partnerships: co-develop an inclusive imaging future

- *Reframe Industry Engagement:* We encourage vendors to see LF-MRI in LMICs as a strategic investment. Supporting open platforms and adaptable, scalable technology allows companies to engage with new user communities, co-develop context-specific solutions, and contribute to a globally relevant data ecosystem that includes disease profiles and environmental exposures historically underrepresented in imaging research.
- *Fund Certification and Deployment:* We call on industry and philanthropic organizations not only to underwrite the certification of open-source systems but also to recognize the broader economic opportunity in doing so. Certified LF-MRI systems unlock trust and adoption in national health systems, enabling scale-up across LMIC markets. By leading in certification, early industry movers can help to catalyse regional manufacturing, stimulate local service ecosystems, and access entirely new markets of tens of millions currently without imaging access. Safe, certified systems help build trust, speed up policy support, and bring together public and private efforts—setting the stage for long-term economic growth while improving health access and equity.
- *Think Beyond the Clinic:* Embrace MRI’s potential in public health research and population science, particularly where high-field imaging is inaccessible.
- *Plan for Future Growth:* As economies and health systems evolve, LF-MRI must be upgradeable and locally guided. LMIC institutions must be positioned as co-architects of this future.

This roadmap is not aspirational—it is actionable. The future of global imaging must be inclusive, sustainable, equitable, and co-developed.

Progress hinges on reimagining neuroimaging as a shared global endeavour, one in which LMIC scientists, engineers, and institutions are not just observers or participants but co-creators of a new imaging paradigm.

Finally, while much of the recent innovation around LF-MRI has centred on neuroimaging—and many commercial platforms are currently head-only systems—we emphasize that LF-MRI is not inherently restricted to brain imaging. Historically, applications at similar or even lower field strengths have included musculoskeletal, abdominal, and prostate imaging, and there is no intrinsic reason why other anatomical targets could not be addressed with modern low-field approaches, given appropriate hardware and acquisition strategies.

## Conclusion

The case for expanding MRI in LMICs has a clinical urgency, but also extends beyond clinical access—it is about creating meaningful representation in global neuroscience and opening doors to scientific opportunity. LF-MRI, designed with sustainability and research empowerment in mind, offers a crucial pathway towards more inclusive and equitable brain science.

The goal of having LF-MRI systems across LMICs must not be to replicate the diagnostic capacity of high-field MRI systems. Instead, it provides an opportunity to establish a new class of programmable, open, and adaptable systems explicitly crafted for the unique needs of LMICs and the evolving priorities of neuroscience research, as well as provide clinical impact to reverse the enormous and growing burden of infectious as well as non-communicable diseases in these regions.

This is not just a matter of providing new technology; it is also about ensuring all scientists and researchers have a voice at the global scientific table of imaging and neuroscience research. LF-MRI, built on open collaboration and sustainability, holds the potential to revolutionize imaging technology worldwide through its innovative challenge of breaking long-held beliefs by the scientific community that more is always better.

However, evidence shows that technological advances alone are insufficient to bring the needed global changes, maintain the quest for innovation, and overcome barriers with limited resources, thereby creating equity in life and science. Technology and innovation must be supported by robust ecosystems, comprising infrastructure, governance, training, ongoing support, and collaborative exchange. We must move beyond one-off donations and the all-too-familiar “drop-and-run” approach that some vendors and charitable organizations have adopted. While well-intentioned, this model is ultimately unsustainable, leading to MRI systems gathering dust in the so-called “medical equipment graveyards” that are seen in hospitals across many LMIC settings. We must learn from these past mistakes.

This is our opportunity to build something undoubtedly unique, sustainable, equitable, widespread, globally relevant, and transformative that will endure and genuinely benefit the scientific and medical community in LMICs. By integrating People, Platforms, and Partnerships, LMIC researchers are well positioned to craft a sustainable, globally relevant model that transcends borders and generations.

Although our focus has been on LMICs, the framework we propose—flexible systems, local empowerment, and open collaboration—has far wider relevance. Rural hospitals, under-resourced urban clinics, and mobile care units in HICs also stand to gain from scalable, affordable, and context-sensitive imaging solutions. Importantly, the lessons learned from implementing LF-MRI in LMIC settings can be directly transferred to these underserved environments in HICs, showing that innovations developed under resource constraints can inform and improve care globally. The challenge we issue is therefore not only about equity, but about reimagining what MRI can be, for everyone.
